# An efficient MPPT techniques for inter-harmonic reduction in grid connected hybrid wind and solar energy systems

**DOI:** 10.1016/j.heliyon.2024.e27312

**Published:** 2024-03-05

**Authors:** Boni Satya Varun Sai, Rupali Mohanty, Satyajit Mohanty, Debashis Chatterjee, C. Dhanamjayulu, Ravikumar Chinthaginjala, Hossam Kotb, Ali Elrashidi

**Affiliations:** a Department of Electrical Engineering, Jadavpur University, Jadavpur, Kolkata, 700032, India; b School of Electrical Engineering, Vellore Institute of Technology, Vellore, 632014, Tamil Nadu, India; c School of Electronics Engineering, Vellore Institute of Technology, Vellore, Tamil Nadu, 632014, India; d Department of Electrical Power and Machines, Faculty of Engineering, Alexandria University, Alexandria, 21544, Egypt; e Electrical Engineering Department, University of Business and Technology, Ar Rawdah, Jeddah, 23435, Saudi Arabia; f Engineering Mathematics Department, Faculty of Engineering, Alexandria University, Alexandria 21544, Egypt

**Keywords:** PV cell modelling, Wind turbine, Solar energy, DFIG and maximum power point tracking

## Abstract

In this work, the operation of photovoltaic system, wind turbine driven doubly fed induction generator along with battery has been observed. Also, a searching space minimization-based artificial bee colony scheme is developed for tracking the maximum power in a doubly fed induction generator-based system. To track maximum power in solar systems, an improved adaptive reference voltage approach has been presented. Several conventional and optimization-based techniques are used by DFIG and photovoltaic systems to get around the non-linearity features in the output parameters. Regarding DFIG, the artificial bee colony method based on searching space minimization can be used to solve the shortcomings of the perturb and observe algorithm. Because of its weather-sensitive nature, it can withstand sudden changes in wind speed. The suggested searching space minimization based artificial bee colony strategy uses a mechanism for determining the range of optimal rotor speed in order to track the maximum power point more quickly. The maximum power point tracking performance of the adaptive reference voltage technique is superior to that of current perturb and observed-based systems. However, a huge processing memory is required in order to track the maximum possible power point. This paper proposes an enhanced maximum power point tracking technique based on adaptive reference voltage that does not require a memory unit. Additionally, despite sudden changes in irradiation conditions, improved adaptive reference voltage can drift-free and reliably monitor the maximum power point. The new adaptive reference voltage technique uses temperature and radiation sensors to identify the region nearest to the maximum power point. This helps the system respond more quickly. The proposed system with searching space minimization based artificial bee colony and improved adaptive reference voltage schemes displays lower inter-harmonic content in grid current compared to perturb and observe scheme. The proposed scheme has been implemented in MATLAB & simulink atmosphere and OPAL-RT displayed satisfactory results.

## 1 Introduction

### 1.1 Hybrid renewable energy system

The WECS and PV systems shares the major share of renewable energy production in modern world [1]. The WECS and PV energy are continuously varying due to the weather dependent nature. Due to this, the alone operation of PV and WECS systems does not guarantee the continuous supply to the consumer. Therefore, it is profitable to operate the wind and PV combination for reliable supply of power [2]. Also, the combined energy from these sources can supply the power to large number of loads. Due to the advancement in technologies, two or many renewable energy sources are operated as HRES [3]. The PV-wind system is further integrated with energy storage systems like the battery, fuel cell etc., in delivering the efficient power supply to the customers [4,5].

The HRES are operated in grid and standalone mode. In grid mode, the PV, wind and battery collectively operate to feed the load. If there is a power deficiency, the load will draw the power from AC grid. The standalone mode is employed in meeting the load requirement of remote areas. Under the temporary unavailability of grid, the grid mode can be treated as standalone. In HRES, the output characteristics of solar and wind are non-linear in nature. Therefore, proper control strategies are required for tracking the maximum power for PV and wind systems [6].

**Fig. 1:**
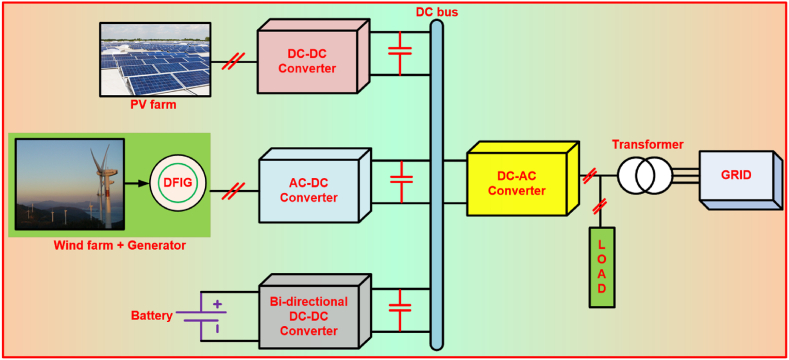
DC coupled HRES system.

**Fig. 2:**
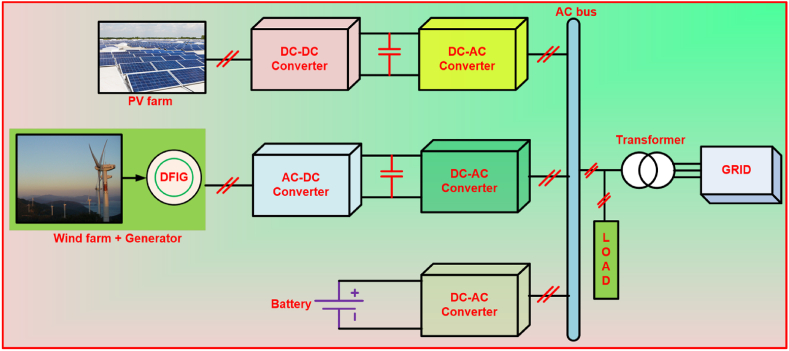
AC coupled HRES system.

The classifications and architecture of HRES are represented in Ref. [7]. The general schemes of converter in HRES are classified into AC-coupled HRES, DC-coupled HRES and Mixed-structure HRES [8]. Fig. 1, represents the DC-coupled HRES system. This system consists of PV system, WECS and battery coupled together with DC-bus. Also, on the other side, a single DC-AC converter is employed in feeding the load. In case of DC-coupled HRES, the MPPT scheme for PV system is applied at DC-DC converter, while for DFIG system it is applied at AC-DC or RSC [9]. Through bi-directional DC-DC converter, battery is associated to DC-bus. Fig. 2 denotes the AC-coupled HRES, where the PV, battery and wind systems are linked to common AC-bus through individual DC-AC converters. Also, for controlling the wind and PV power, MPPT schemes are employed. There is mixed HRES, in which displays advantages of the both AC and DC coupled schemes [10].

A PSO–BPSO Technique, Particle Swarm Optimization Algorithm and Two-Stage Optimization are employed in sizing of the HRES [[11], [12], [13]]. Along with the sizing, the economic assessment of the HRES implementation is defined in Ref. [14]. The wind/PV along with battery/supercapacitor-based systems are defined in Refs. [15,16]. The HRES can be operated in grid connected and standalone modes. It is observed that most of the HRES are operated in the standalone mode. In Ref. [17], there is system established for the standalone microgrid operation. The enhancement of the standalone hybrid energy system in the regard of power quality is projected in Ref. [18]. [19], shows the results of a sensitivity study for a freestanding hybrid system for a rural healthcare facility. The grid connected HRES are discussed in Ref. [20]. From Refs. [[21], [22], [23]], the operating properties of the system can be seen to be significantly influenced by the MPPT schemes. Also, it is known that, MPPT schemes plays key role in producing inter-harmonics into grid [24]. There is a vacuum left for the study of inter-harmonics with respect to wind case. The inter-harmonics are mainly caused due to the not so effective tracking characteristics such as greater, settling time and steady state error. In the proposed schemes, advanced MPPT schemes such SSM-ABC and IARV MPPT methods are employed. The proposed system able to address the tracking characteristics, thus reducing the introduction of inter-harmonics into the system. In this work, improved MPPT techniques are developed for wind and PV systems to display the desired characteristics.

### 1.2 MPPT schemes in photovoltaic system

For the optimum usage of available PV panel power, different MPPT schemes has been proposed [25,26]. The MPPT methods are classified into two classes, they are conventional and advanced MPPT schemes. The conventional schemes are simple and easy real time implementable compared to advanced MPPT schemes like ANN and optimization etc. Therefore, the conventional schemes are most considered for industrial purpose. P&O algorithm is the widely employed method among the available conventional MPPT schemes, due to its simple construction and efficient MPP detection [27,28]. The output power fluctuations with abrupt changes in radiation levels are shown by the P&O method. By utilizing an additional current sensor in the P&O system, modified P&O can eliminate output fluctuations caused by abrupt changes in solar irradiation [29].

The CV MPPT is economical as it requires a single voltage sensor in tracking maximum power [30,31]. Low tracking efficiency in lower irradiation circumstances is CV MPPT's main drawback. In order to overcome the limitations of the CV technique, irradiation and temperature sensors are used in the adaptive reference voltage MPPT. The requirement of huge processing memory in tracking MPP is the major setback for ARV MPPT scheme [32].

In [33,34], inter-harmonic elimination is dealt with using CV and P&O MPPT with variable sampling rate. The MPPT parameters, however, such as efficiency and oscillations over rapid variations in solar irradiation, are not addressed. So, there is a higher necessity of a MPPT scheme for reducing the harmonic injection into the grid along with maintaining good MPP tracking characteristics.

The IARV MPPT algorithm is used in this study to track the maximum power output of PV systems. Comparing IARV to P&O-based schemes, it exhibits better MPPT characteristics. Unlike CV scheme, it displays better tracking efficiency at lower irradiation cases. No memory unit is necessary for the IARV scheme to track MPP. In comparison to the ARV plan, the suggested solution is cost-effective and simple to execute in real time. Also, IARV displays low inter-harmonic content compared to Refs. [33,34], along with good dynamic characteristics.

### 1.3 MPPT schemes in WECS

The production of wind energy commonly makes use of the DFIG. Static power converter production in DFIG is more inexpensive than in PMSG [35]. Non-linearity parameters in DFIG are evident in the mechanical power and rotor speed characteristics. Therefore, MPPT schemes are implemented in DFIG for extracting maximum power. P&O algorithm displays abrupt variation in tracked DFIG MPP power under fluctuating wind conditions. Under both small and large increments in rotor speeds, the P&O approach produces increased settling time and steady state oscillations [36]. Therefore, both these cases may inject interharmonics into the grid. Many optimization-based schemes are introduced to eliminate the shortcomings of P&O scheme.

The novel RPO-FOSMC method described in Ref. [37] leads to good dynamic characteristics, it undergoes oscillations under sudden change in wind speed case. A four sectors operation-based P&O method is proposed in Ref. [38]. Instead of faster tracking [38], experiences higher response time related to the large step P&O algorithm. A fixed-time control method is employed in Ref. [39], for MPP tracking of DFIG system. The GGWO technique in Ref. [40] is not expanded for GSC. Optimization-based schemes are introduced overcome the disadvantages of P&O based schemes in [[41], [42], [43], [44], [45], [46]].

The settling time required for optimization-based schemes in tracking MPP is higher. Among the existing methods, PSO is easier to implement in real time [47]. CS algorithm exhibits better operating features compared to PSO [48,49]. In Ref. [50], artificial bee colony based MPPT scheme has been proposed, it displays improved energy returns compared to existing PSO scheme. In this paper, an SSM-ABC is introduced as a modified form of ABC. In proposed scheme the tracking time is considerably decreased by employing searching space minimization mechanism.

The optimization-based schemes are employed to extract the parameters of solar panels [51,52]. In the proposed scheme, extraction of parameters is not majorly focused at. Even though the mentioned schemes are able to produce the accurate output characteristics, they add to the mathematical burden. The proposed scheme mainly focusing on a system with mathematical burden. Therefore, the parameter extraction-based techniques are not employed in the proposed system. There are many challenges existing with respect to the PV integration with grid [[53], [54], [55], [56], [57]]. In the proposed system, PV system and wind assist each other to maintain the constant DC link voltage, further providing the grid stability. The proposed system can be used to deliver the power to remote hilly areas, where it is difficult to provide the power using regular transfer methods.

Overall, HRES along with proposed IARV and SSM-ABC schemes has been implemented for a PV system of 12 KW, DFIG of 10 KW and battery of 5 KW in MATLAB & Simulink atmosphere and OPAL-RT. Additionally, when compared to the system using the P&O scheme, the system using the suggested MPPT schemes showed enhanced dynamic characteristics and a lower inter-harmonic content. The paper is formulated as system configuration in section 2, proposed MPPT control strategies for HRES in section 3, Results and discussions in section 4, conclusion in section 5.

## 2 System configuration

### 2.1 PV modelling

**Fig. 3:**
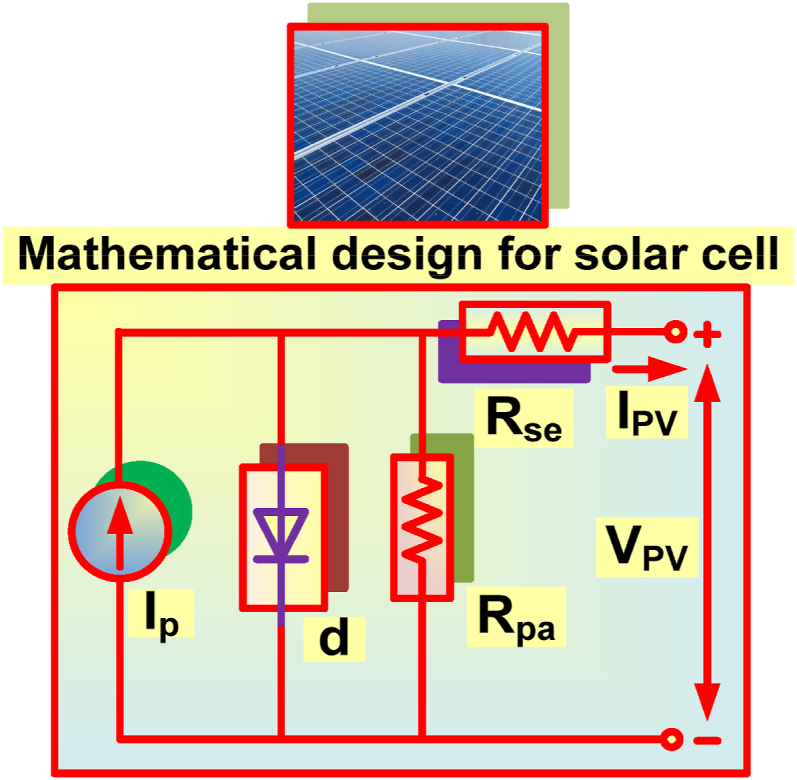
Solar cell representation.

Single diode model is mostly employed for solar cell modelling due to its accuracy in producing the output characteristics [29]. Fig. 3, represents the solar cell circuit employing single diode model. Using Fig. 3, the mathematical relation amongst I_PV_ and V_PV_ can be formulated as (1).

(1) $$I_{PV}=I_{PV}-I_{o}\left(e^{\frac{q\left(V_{PV}+R_{se}I_{PV}\right)}{AkT}}-1\right)-\frac{V_{PV}+R_{se}I_{PV}}{R_{pa}}$$

In terms of temperature and irradiation, the I_sho_ and V_ope_ are expressed as (2 & 3),

(2) $$I_{sho}=I_{sho,ref}\left[1+\alpha \left(T-T_{ref}\right)\right]\frac{G}{G_{ref}}$$

(3) $$V_{ope}=V_{ope,ref}\left[1+a\times \ln\frac{G}{G_{ref}}+T_{\beta }\left(T-T_{ref}\right)\right]$$

**Fig. 4:**
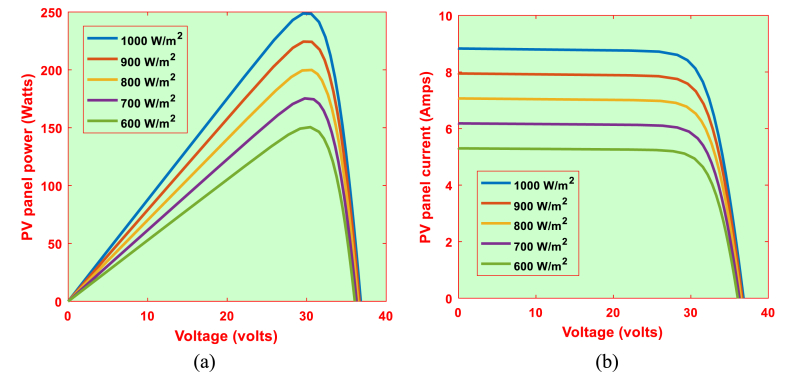
Output characteristics of 1S–1P configuration. (a) P–V characteristics (b) I–V characteristics.

The solar panel manufacturer data is shown in Table-2. P–V and I–V characteristics for the solar panel under consideration are shown in Fig. 4. Using Fig. 4a & b, the V_ope_, I_sho_, MPP voltage, MPP current and MPP power values are represented in Table-1 (see Table 3).

**Table 1:** P_m_, V_m_, I_m_, V_ope_and I_s_for 1S–1P Configuration.

G (W/m^2^)	P_M_ (KW)	V_M_ (Volts)	I_M_ (Amps)	V_ope_ (Volts)	I_sho_ (Amps)
600	150.54	30.44	4.95	36.05	5.30
700	175.64	30.04	5.85	36.28	6.19
800	200.40	29.97	6.69	36.47	7.07
900	224.84	29.82	7.54	36.64	7.95
1000	249.1	30.01	8.3	36.79	8.83

**Table 2:** System parameters.

WT driven DFIG parameters	
Peak power	10 KW
Pole pair	4
Peak voltage	400V
Stator resistance	0.2147 Ω
Rotor resistance	0.2205 Ω
Stator leakage inductance	0.991 mH
Magnetizing inductance	64.19 mH
Rotor leakage inductance	0.991 mH
Turns ratio	0.34
Blade radius	3.45 m
Air density	1.225 $kg/m^{3}$
Damping Coefficient	0.01 Nm.s/rad
Inertia constant	2 s
Optimal Tip speed ration	7.2
Maximum power coefficient	0.44
Unknown coefficients (C_1_–C_7_)	C_1_ = 0.5176, C_2_ = 116, C_3_ = 0.4, C_4_ = 5, C_5_ = 21, C_6_ = 0.0068, C_7_ = 0.08
Solar panel characteristics (TP250MBZ)	
MPP Power	249 W
MPP Voltage	30 V
MPP Current	8.3 amps
Short Circuit Current	8.83 amps
Open Circuit Voltage	36.8 V
Temperature coefficient of I_sho_	0.064%/^0^C
Temperature coefficient of V_ope_	−0.33%/^0^C
Storage system	
Rated battery capacity	75 Ah
Rated battery Voltage	12 V

**Table 3:** P_m_, V_m_, I_m_, V_ope_and I_s_for 24S–2P Configuration.

G (W/m^2^)	P_M_ (KW)	V_M_ (Volts)	I_M_ (Amps)	V_ope_ (Volts)	I_sho_ (Amps)
600	7.23	730.56	9.9	865.2	10.6
700	8.43	720.96	11.7	870.72	12.38
800	9.62	719.28	13.38	875.28	14.14
900	10.79	715.68	15.08	879.36	15.9
1000	11.96	720.24	16.6	882.96	17.66

### 2.2 Wind turbine modelling

**Fig. 5:**
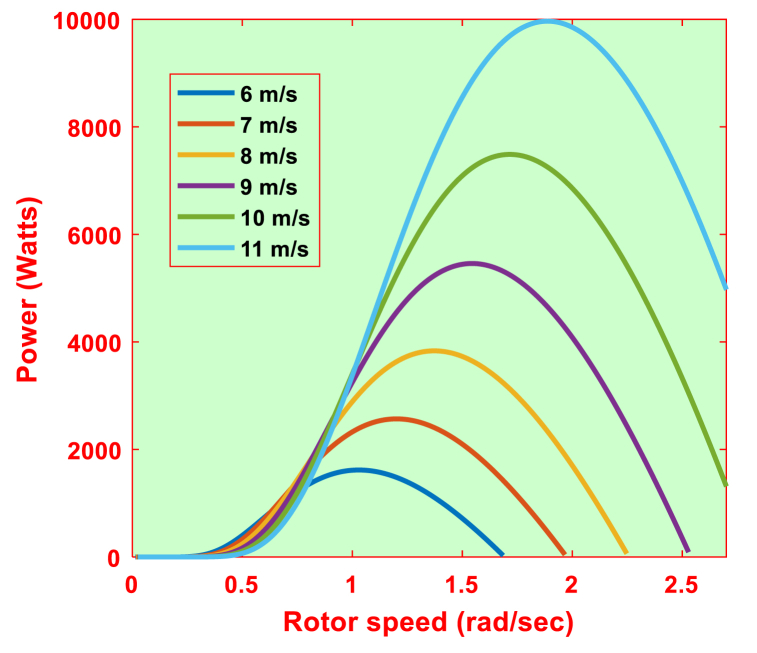
Power vs rotor speed characteristics at various wind speed levels.

The mechanical power generated by WT is defined as (4). The $\lambda_{i}$ can be evaluated by employing β and λ as (5)–(6). The λ can be measured using rotor speed as shown in (7). At the MPP, (7) can be modified as (8). The wind turbine characteristics are represented in Table-2. Fig. 5 shows the power-rotor speed characteristics for the turbine parameters taken into consideration at various wind speeds.

(4) $$P_{m}=\frac{1}{2}\rho A_{r}C_{p}\left(\beta ,\lambda \right)V_{w}^{3}$$

(5) $$C_{p}=C_{1}\left(\frac{C_{2}}{\lambda_{i}}-C_{3}\beta -C_{4}\beta^{C_{5}}-C_{6}\right)\left(e^{C_{7}/\lambda_{i}}\right)$$

(6) $$\lambda_{i}=\frac{1}{\lambda +0.02\beta }-\frac{0.003}{1+\beta^{3}}$$

(7) $$\lambda =\frac{\omega_{m}R}{V_{w}}$$

(8) $$\lambda_{opt}=\frac{\omega_{opt}R}{V_{w}}$$

### 2.3 DFIG modelling

**Fig. 6:**
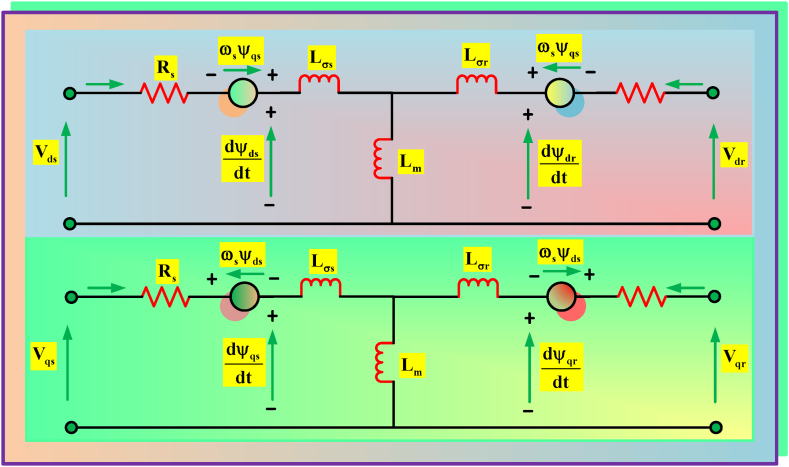
dq-reference frame for DFIG design.

In Fig. 6, the DFIG modeling using the dq-reference frame is shown. (9, 10, 11 &12) [58], is a representation of the rotor and stator voltage equations.

(9) $$v_{ds}=R_{s}i_{ds}+\frac{d\psi_{ds}}{dt}-\omega_{s}\psi_{ds}$$

(10) $$v_{qs}=R_{s}i_{qs}+\frac{d\psi_{qs}}{dt}-\omega_{s}\psi_{qs}$$

(11) $$v_{dr}=R_{r}i_{dr}+\frac{d\psi_{dr}}{dt}-\omega_{r}\psi_{dr}$$

(12) $$v_{qr}=R_{r}i_{qr}+\frac{d\psi_{qr}}{dt}-\omega_{r}\psi_{qr}$$

The flux generated in DFIG are denoted by employing (13, 14, 15 &16).

(13) $$\psi_{ds}=L_{s}i_{ds}+L_{m}i_{dr}$$

(14) $$\psi_{qs}=L_{s}i_{qs}+L_{m}i_{qr}$$

(15) $$\psi_{dr}=L_{m}i_{ds}+L_{r}i_{dr}$$

(16) $$\psi_{qr}=L_{m}i_{qs}+L_{r}i_{qr}$$

The $T_{em}$ is denoted as (17),

(17) $$T_{em}=\frac{3}{2}p\frac{L_{m}}{L_{s}}\left(\psi_{qs}i_{dr}-\psi_{ds}i_{qr}\right)$$

### 2.4 Overall system configuration

**Fig. 7:**
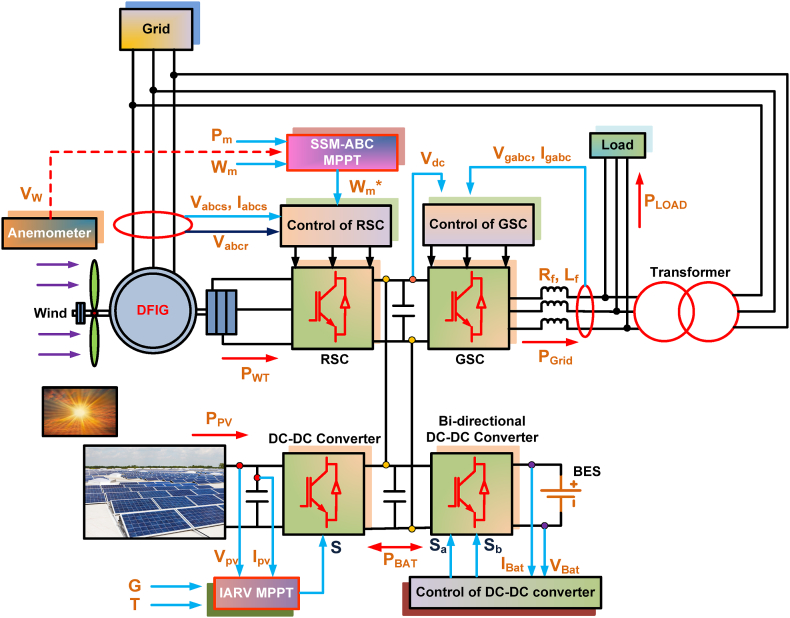
Block diagram for proposed HRES system.

The proposed system is developed while taking into account the DC-coupled HRES system. Fig. 7 represents the proposed system's overall block diagram. The DFIG, PV and battery system are operated together to supply the continuous supply to the load. The proposed system consists of DFIG system with RSC, PV configuration with DC-DC converter and Battery with bi-directional DC-DC converter. These all-coupled systems are connected to GSC through a DC link capacitor. The suggested system's parameters are denoted in Table-2.

## 3 PROPOSED MPPT control strategies for HRES

In this section, the proposed MPPT schemes for PV and wind systems are thoroughly discussed. Also, the idea behind the development of proposed schemes is properly defined.

### 3.1 Proposed IARV MPPT scheme for PV system

**Fig. 8:**
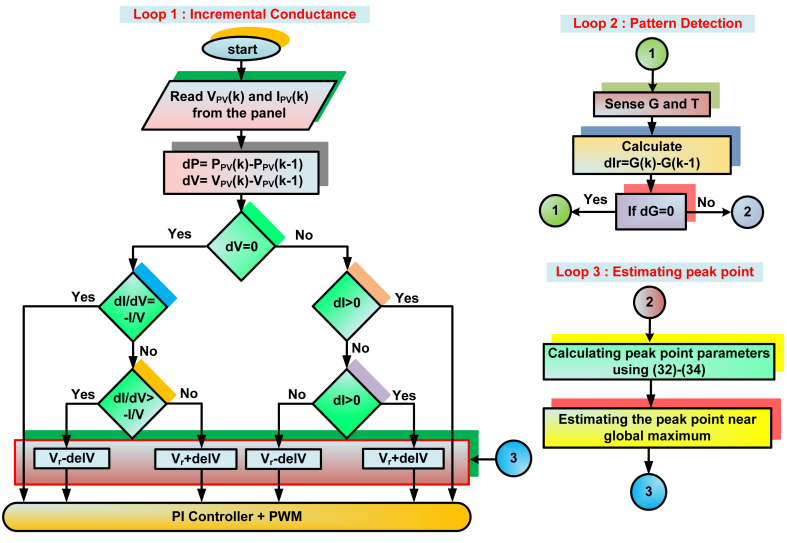
Proposed IARV MPPT scheme.

The algorithm for the suggested IARV method is shown in Fig. 8. The closest point to MPP is calculated using the specified peak detection criteria in the proposed method. This nearest point is employed as the starting point for tracking in incremental conductance algorithm. The proposed IARV method is the modified version of Adaptive reference voltage (ARV) MPPT method. In the proposed method, mathematical modelling has been developed to estimate the MPP with respect to the irradiation variation. As a result, unlike current traditional MPPT systems, quicker MPP tracking is achieved in proposed scheme without any initial perturbation. Unlike ARV method, IARV MPPT scheme does not require any memory unit to track the MPP, resulting in less computational cost. These things differentiate the IARV from other traditional approaches.

#### 3.1.1 Relation between MPP values for single panel and aS-bP configuration under uniform shading

**Fig. 9:**
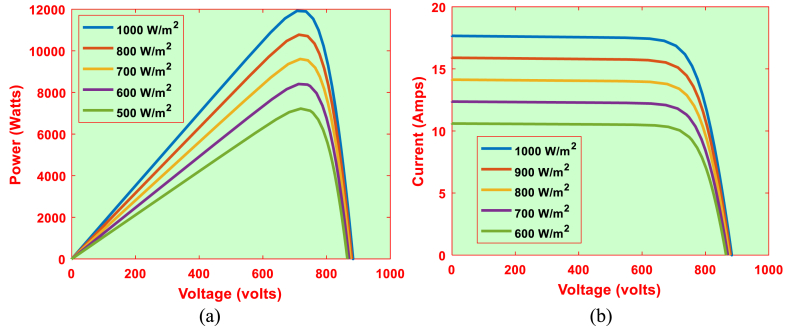
Output characteristics of 24S–2P configuration. (a) P–V characteristics (b) I–V characteristics.

For finding the relation between MPP values, aS-bP configuration is considered in the proposed system, where a = 24 and b = 2. The P–V and I–V curves for the considered PV system is represented in Fig. 9. Using Fig. 9a & b, the V_ope_, I_sho_, MPP voltage, MPP current and MPP power values are represented in Table-3.

**Table 4:** Tracking time and efficiency comparison of IARV MPPT scheme with P&O based schemes.

Properties	P&O	MP&O	IARV
Efficiency (%)	98.14	98.37	99.09
Tracking time (ms)	26.2	26.18	14.5

By observing Table-1 and Table-3, when a single panel and a 24S–2P arrangement are used, the relationship between the MPP voltage values can be found to be (18, 19, 20, 21 & 22),

(18) $${\left({V_{M}|}_{24S-2P}\right)}_{600}\approx 24\times {\left({V_{M}|}_{1S}\right)}_{600}$$

(19) $${\left({V_{M}|}_{24S-2P}\right)}_{700}\approx 24\times {\left({V_{M}|}_{1S}\right)}_{700}$$

(20) $${\left({V_{M}|}_{24S-2P}\right)}_{800}\approx 24\times {\left({V_{M}|}_{1S}\right)}_{800}$$

(21) $${\left({V_{M}|}_{24S-2P}\right)}_{900}\approx 24\times {\left({V_{M}|}_{1S}\right)}_{900}$$

(22) $${\left({V_{M}|}_{24S-2P}\right)}_{1000}\approx 24\times {\left({V_{M}|}_{1S}\right)}_{1000}$$

Similar to that, it is possible to determine the relationship between the MPP current values for the 1S and 24S–2P configurations as follows (23, 24, 25, 26 & 27),

(23) $${\left({I_{M}|}_{24S-2P}\right)}_{600}\approx 2\times {\left({I_{M}|}_{1S}\right)}_{600}$$

(24) $${\left({I_{M}|}_{24S-2P}\right)}_{700}\approx 2\times {\left({I_{M}|}_{1S}\right)}_{700}$$

(25) $${\left({I_{M}|}_{24S-2P}\right)}_{800}\approx 2\times {\left({I_{M}|}_{1S}\right)}_{800}$$

(26) $${\left({I_{M}|}_{24S-2P}\right)}_{900}\approx 2\times {\left({I_{M}|}_{1S}\right)}_{900}$$

(27) $${\left({I_{M}|}_{24S-2P}\right)}_{1000}\approx 2\times {\left({I_{M}|}_{1S}\right)}_{1000}$$

#### 3.1.2 (see ) Table 4Proposed peak point detection for aS-bP configuration under uniform shading

By observing (18)–(27), It should be noted that using MPP values from a single solar panel, series-parallel setups can be formed. The proposed peak point detection can be explained further using three steps.

Step-1 For the sensed irradiation and temperature values, calculate the I_sho_ and V_ope_ using (2), (3).

Step-2 Using the I_sho_ and V_ope_, the MPP current values of individual solar panels can be calculated as,

(28) $${\left({V_{M}|}_{1S}\right)}_{G}\approx K_{1}\times {\left({V_{ope}|}_{1S}\right)}_{G}$$

(29) $${\left({I_{M}|}_{1S}\right)}_{G}\approx K_{2}\times {\left({I_{sho}|}_{1S}\right)}_{G}$$

Where K_1_ and K_2_ are calculated as,

(30) $$K_{1}\approx \frac{{\left({V_{M}|}_{1S}\right)}_{STC}}{{\left({V_{ope}|}_{1S}\right)}_{STC}}$$

(31) $$K_{2}\approx \frac{{\left({I_{M}|}_{1S}\right)}_{STC}}{{\left({I_{sho}|}_{1S}\right)}_{STC}}$$

Step-3 By employing (28, 29, 30 & 31), the MPP values for aS-bP configuration can be calculated as,

(32) $${\left({V_{M}|}_{24S-2P}\right)}_{G}\approx a\times {\left({V_{M}|}_{1S}\right)}_{G}$$

(33) $${\left({I_{M}|}_{24S-2P}\right)}_{G}\approx b\times {\left({I_{M}|}_{1S}\right)}_{G}$$

(34) $${\left({P_{M}|}_{24S-2P}\right)}_{G}\approx {\left({V_{M}|}_{1S}\right)}_{G}\times {\left({I_{M}|}_{1S}\right)}_{G}$$

By using (32), (33), (34), the nearest point to MPP is measured. This point is employed in setting the initial point of searching for incremental conductance method. The estimating peak mechanism helps IARV scheme to settle faster related to P&O based algorithms. Additionally, compared to the P&O approach, the suggested method exhibits less volatility when the irradiation changes suddenly.

### 3.2 Proposed SSM-ABC scheme for WECS

**Fig. 10:**
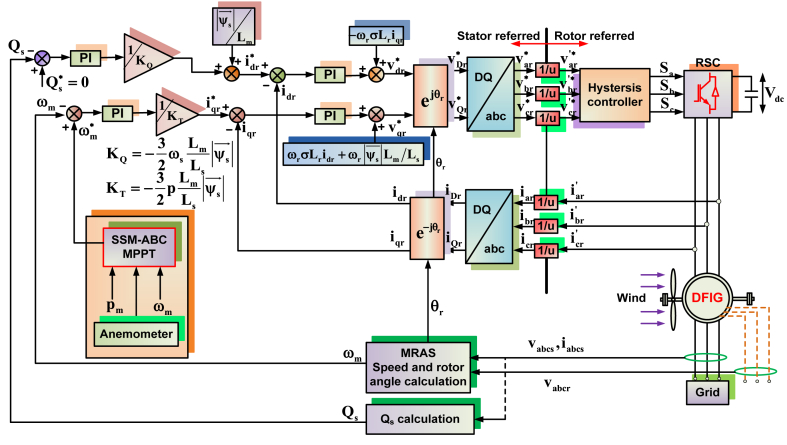
Proposed control scheme for RSC in DFIG system.

The upgraded MPPT methods are the primary emphasis of the suggested system. So, the proposed scheme is well addressed with RSC side, while GSC converter control is similar to that of [1]. Fig. 10 denotes the overall RSC control system along with proposed SSM-ABC method. In this work, SSM-ABC method has been developed for tracking maximum power in DFIG system. It mainly focuses on reducing the minimization of searching space in tracking MPP. As shown in Fig. 10, the position and speed estimation of rotor is done by employing MRAS control strategy [59]. Wind speed sensor is used to optimize the searching area for making the algorithm to search the MPP faster. SSM-ABC uses the steady state error reducing strategy similar to that of SSM-PSO scheme [58].

**Fig. 11:**
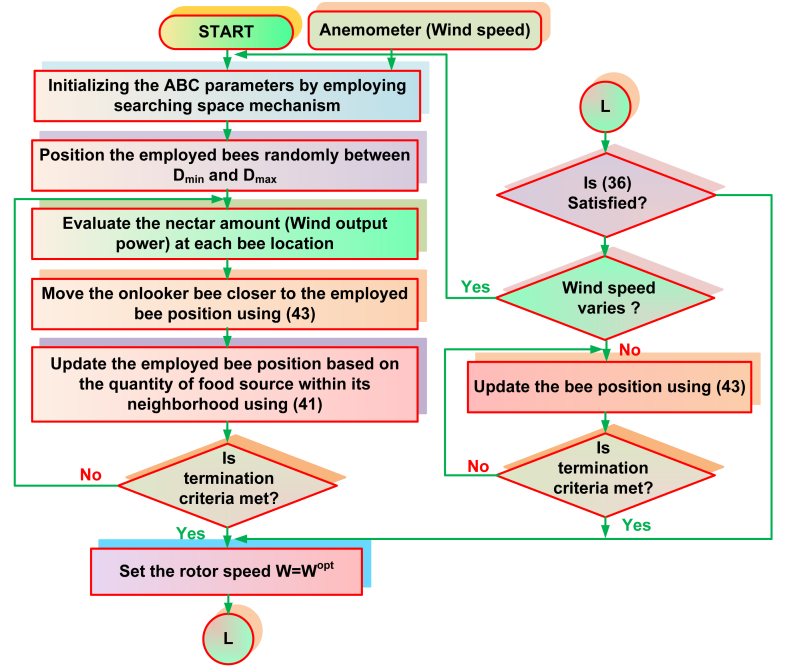
Proposed SSM-ABC algorithm.

The proposed SSM-ABC algorithm has been represented in Fig. 11. Before describing the step wise SSM-ABC employment, Np number of bees has been considered as a solution vector for rotor speeds (35).

(35) $$\begin{array}{c} x_{i}^{k}=\omega_{g}=\left[\omega_{1},\omega_{2},\omega_{3},......,\omega_{j}\right]\\ j=1,2,3,4,......................N_{P} \end{array}$$

The following is a definition of the suggested system's objective function (36),

(36) $$P\left(\omega_{i}^{k}\right)-P\left(\omega_{i}^{k-1}\right)=f_{k}$$

The function $f_{k}$ can be determined subjected to $P\left(\omega_{i}^{k}\right)>P\left(\omega_{i}^{k-1}\right)$, where $P\left(\omega_{i}\right)$ can be obtained using (4)–(7).

For SSM-ABC formulation, the DFIG output power is considered as nectar amount. Additionally, in the SSM-ABC approach, the decision variable designated as the position of the food supply is the rotor speeds. In this proposed scheme, the tracking time is minimized by reducing the searching space. The step wise operation of SSM-ABC scheme in tracking MPP is denoted below.

Step-1 Proposed initialization

Initialize the N_P_, wherein half the bees are thought to be working bees and the other half to be spectator bees. All of these bees are initially positioned utilizing different food source positions (37).

(37) $$\begin{array}{c} x_{i}=\omega_{\min}+\frac{\left(i-1\right)\left[\omega_{\max}-\omega_{\min}\right]}{N_{p}-1}\\ i=1,2,3,4,......................,N_{p} \end{array}$$

Where $\omega_{\max}$ and $\omega_{\min}$ are calculated by employing proposed initialization strategy.

Anemometer is used to calculate the optimum rotor speed value as (38). Further, the measured optimum rotor speed value is used set $\omega_{\max}$ and $\omega_{\min}$ values by employing (39) and (40). This initialization part is helpful in placing the bees nearest to MPP.

(38) $$\omega_{opt}=\frac{\lambda_{opt}V_{w}}{R}$$

(39) $$\omega_{\max}=\omega_{opt}+\in$$

(40) $$\omega_{\min}=\omega_{opt}-\in$$

Step-2 Estimating the Quantity of the Nectar

The amount of output power for each rotor speed reference is calcualted using mathematical formulation in simulation. The measured nectat amount is used to decide to assign the employer and onlooker bees.

Step-3 Finding new food source position

The process of searching MPP for DFIG system is done in two phases, where first phase is for employed bees and second phase is for onlooker bees. Employed bees update their positions by employing (41).

(41) $$\begin{array}{c} {x_{i}}^{k+1}={x_{i}}^{k}+\gamma \left({x_{i}}^{k}-{x_{j}}^{k}\right)\\ j\in \left(1,2,...............,\frac{N_{P}}{2}\right) \end{array}$$

Where γ values are in the levels of [-1, 1]. At their new places, the employed bees now measure the amount of food source. If the quantity at new locations is higher, then the employed bees stays at the updated position. Otherwise, the employed bees return to the old position as (42).

(42) $${x_{i}}^{k+1}={x_{i}}^{k}$$

The onlooker bees dances towards the employed bee location where the quantity if food source is higher using (43).

(43) $${x_{i}}^{k+1}={x_{hi}}^{k}+\frac{\gamma \left[\omega_{\max}-\omega_{\min}\right]}{\frac{N_{p}}{2}-1}$$

Where X_hi_ denotes the food source location with high nectar quantity.

Step-4 Termination Conditions:

If there is no further increase in the amount of DFIG output power after five consecutive iterations, termination of the process begins. The present position is employed as optimum rotor speed for tracking MPP. If termination state is not happened, system will go to step-2.

Step-5 Reinitializing the process:

If there is a variation in wind speed, the process will restart the bee initialization. This wind speed variation can be observed by anemometer.

**Fig. 12:**
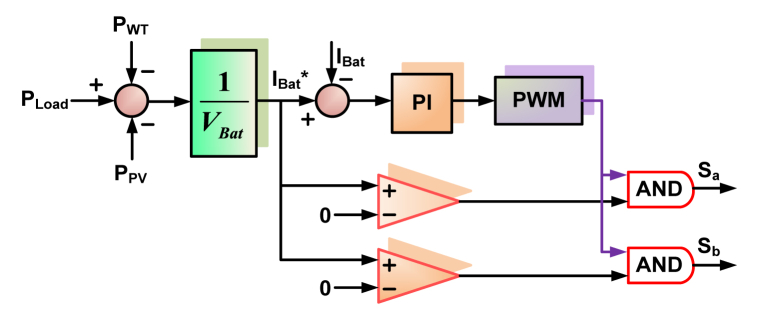
Battery control strategy.

To address the drawbacks of the current P&O scheme, the SSM-ABC proposal is made. In comparison to the SS-P&O system, the proposed scheme shows a shorter settling time, and it also shows a lower steady state oscillation than the LS-P&O scheme [36]. In comparison to other optimization-based techniques now in use, the proposed system is also simple to implement. The battery control strategy for the considered system is represented in Fig. 12. This control strategy is similar to that of the technique used in Ref. [1].

## 4 Results and discussion

**Fig. 13:**
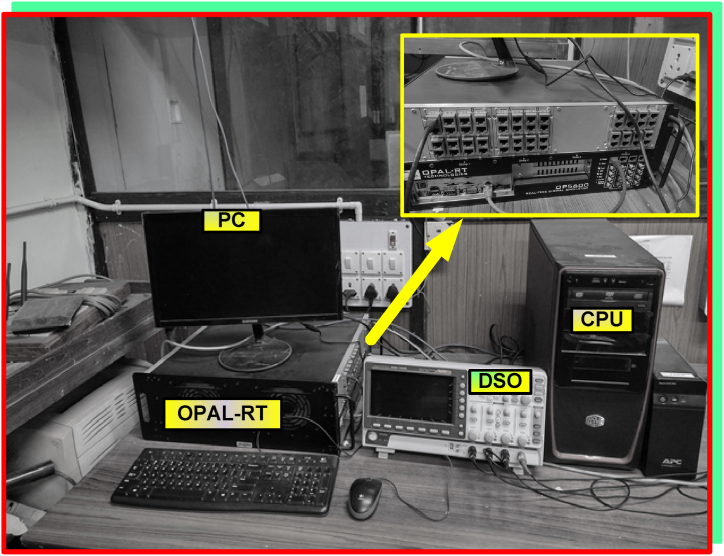
OPAL-RT setup in laboratory.

The system with proposed IARV and SSM-ABC schemes are implemented in MATLAB & simulink atmosphere and OPAL-RT. The OPAL-RT setup in laboratory is represented in Fig. 13.

### 4.1 Analysis of proposed IARV scheme

**Fig. 14:**
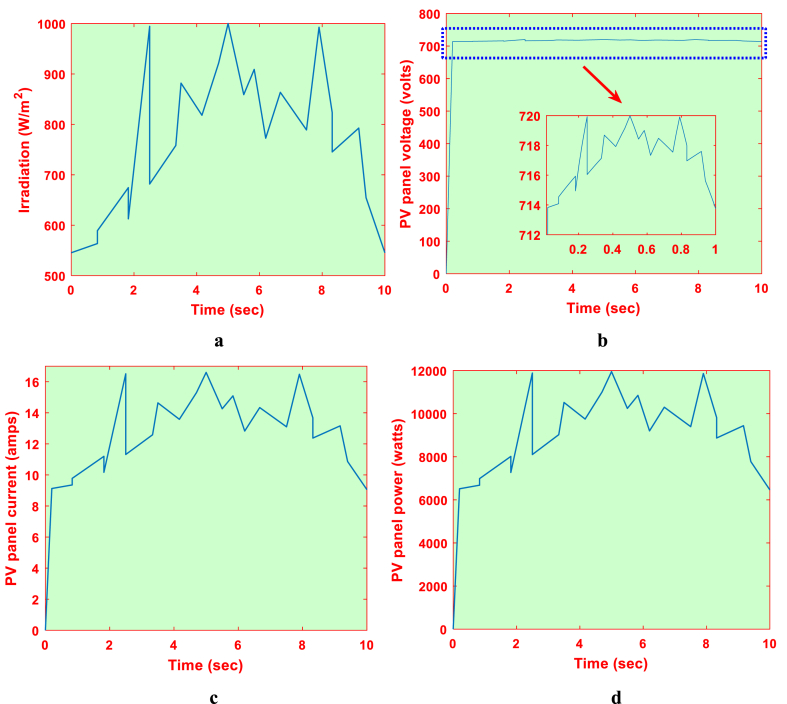
PV system with proposed IARV MPPT (a) Irradiation, (b) Voltage, (c) Current and (d) Power.

As depicted in Fig. 8, the PV system is run for the IARV MPPT algorithm. Additionally, as illustrated in Fig. 14a, the proposed system is used for the irradiation levels. PV panel voltage, current, and power are depicted in Fig. 14b, c & 14c, respectively, for the irradiation pattern under consideration. As seen in Fig. 14d, the suggested IARV MPPT technique may effectively find the MPP. IARV approach is able to track the MPP faster compared to P&O and MP&O schemes because of its close MPP detection strategy [29]. The tracking time according to the IARV MPPT technique is 14.5 ms. The suggested system, unlike P&O, does not exhibit any drift with a sudden shift in irradiation. The improved MPPT efficiency is demonstrated by the proposed IARV MPPT scheme of 99.09%.

**Fig. 15:**
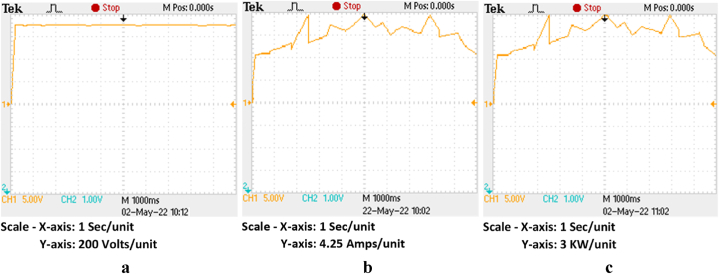
Proposed IARV MPPT in OPAL-RT (a) voltage, (b) current and (c) power.

Although [33] decreases inter-harmonics, its main drawback is tracking inefficiency in lower irradiation circumstances. Along with preserving strong dynamic properties, the IARV scheme can show increased effectiveness in all conceivable irradiation situations. The suggested plan has been put into practice in OPAL-RT. Fig. 15a, b & 15c, respectively, show the voltage, current, and power of each individual PV panel. Table-4 compares the tracking effectiveness and speed of the IARV and P&O-based methods. From this, it can be seen that, other from drift avoidance, the P&O and MP&O algorithms' operational characteristics are remarkably similar (see Table 5).

**Table 5:** Assessment of SSM-ABC with P&O scheme.

Algorithms	Settling time	Oscillations	Efficiency
LS-P&O [36]	77 ms	1.2	–
SS-P&O [36]	>1300 ms	0.05	87.11%
SSM-PSO	<350 ms	0.001	92.01%
Proposed SSM-ABC	<350 ms	0.001	92.12%

### 4.2 Analysis of proposed SSM-ABC scheme

**Fig. 16:**
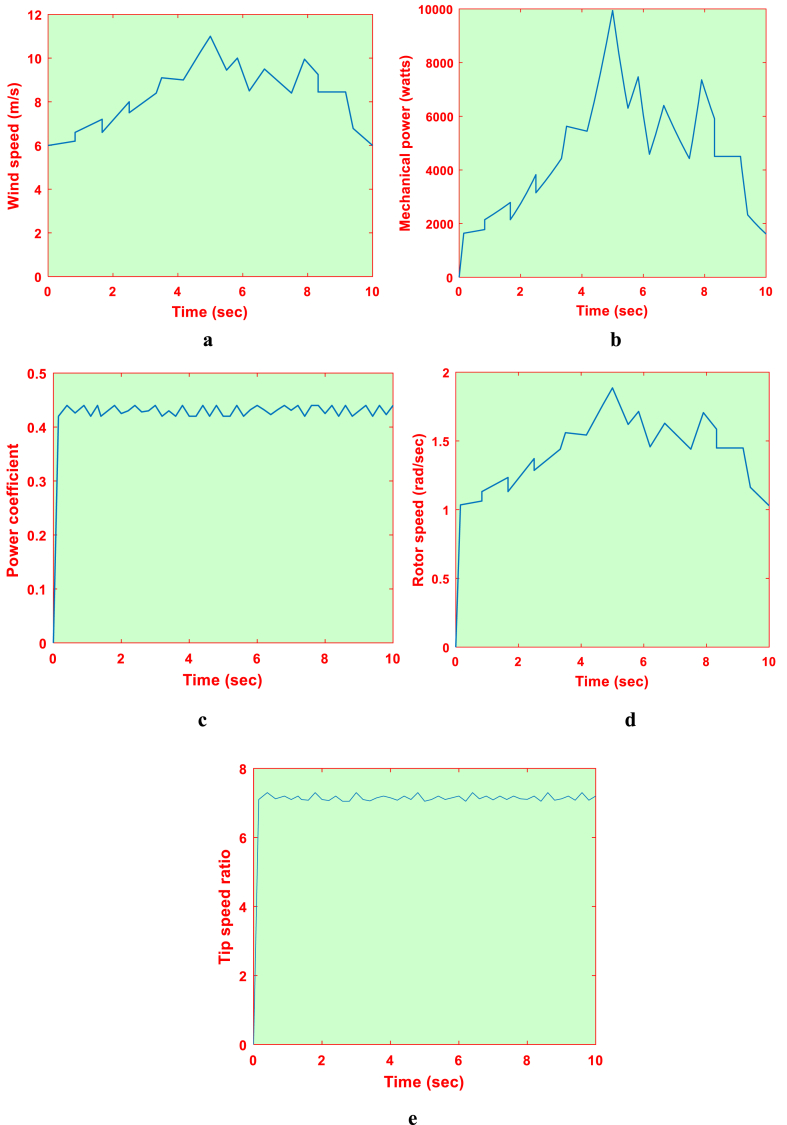
DFIG system with proposed SSM-ABC MPPT (a) wind speed profile, (b) Power, (c) Power coefficient, (d) Rotor speed and (e) Tip speed ratio.

The DFIG system is operated with the proposed SSM-ABC scheme for MPPT. The system is functioned for the wind speed profile as represented in Fig. 16a. The DFIG parameters such as mechanical power, power coefficient, rotor speed and tip speed ratio respectively are represented in Fig. 16b, c, 16d & 16e respectively. From this it is noted that the proposed SSM-ABC scheme is able to detect the MPP effectively with low settling time. Also, it is noted that the SSM-ABC does not deviate or fluctuate under the random variation of wind speed.

**Fig. 17:**
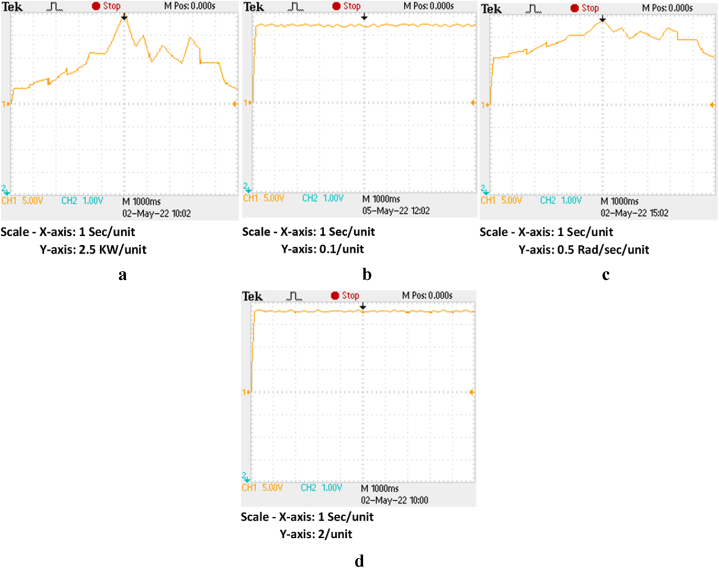
Proposed SSM-ABC in OPAL-RT (a) Power, (b) Power coefficient, (c) Rotor speed and (d) Tip speed ratio.

In comparison to SS-P&O (>1300 ms), the suggested approach demonstrates a faster response time (<350 ms). Additionally, compared to the LS-P&O, the SSM-ABC exhibits steady state oscillations that are 0.001 rad/s lower. The suggested plan has been put into practice in OPAL-RT. Fig. 17a, b, 17c & 17d show the corresponding mechanical power, power coefficient, rotor speed, and tip speed ratio. Table-5 compares the proposed SSM-ABC scheme to the P&O-based schemes. When compared to the P&O scheme, the proposed method has an efficiency improvement of 92.12% (see Table 6).

**Table 6:** Inter-harmonic comparison between P&O based system and proposed system.

FREQUENCY (Hz)	P&O	Proposed system	FREQUENCY (Hz)	P&O	Proposed system
0	0.21	0.15	51.72	4.61	4.3
1.72	0.27	0.19	53.45	2.11	1.38
3.45	0.3	0.2	55.17	1.94	1.5
5.17	0.29	0.2	56.89	1.89	1.2
6.89	0.32	0.21	58.62	1.44	0.94
8.62	0.32	0.22	60.34	1.62	1.02
10.34	0.35	0.22	62.06	1.21	0.72
12.07	0.35	0.24	63.79	1.41	0.87
13.79	0.39	0.24	65.52	1.07	0.61
15.52	0.41	0.27	67.24	1.23	0.75
17.24	0.44	0.26	68.97	0.98	0.55
18.97	0.47	0.31	70.69	1.08	0.66
20.69	0.49	0.28	71.41	0.91	0.51
22.41	0.55	0.36	74.14	0.97	0.59
24.14	0.54	0.31	75.86	0.85	0.48
25.86	0.65	0.42	77.58	0.87	0.53
27.59	0.61	0.34	79.31	0.8	0.46
29.31	0.78	0.49	81.03	0.79	0.48
31.03	0.68	0.38	82.76	0.76	0.44
32.76	0.94	0.59	84.48	0.73	0.44
34.48	0.78	0.45	86.21	0.72	0.42
36.21	1.14	0.72	87.93	0.68	0.41
37.93	0.92	0.55	89.66	0.68	0.4
39.66	1.38	0.88	91.38	0.64	0.38
41.38	1.15	0.77	93.1	0.65	0.39
43.01	1.71	1.09	94.83	0.61	0.36
44.83	1.65	1.33	96.55	0.62	0.37
46.55	2.01	1.31	98.28	0.58	0.35
48.28	4.31	4.13	100	0.59	0.36
			**AVERAGE**	**0.991**	**0.672**

### 4.3 Comparison of inter-harmonic content

**Fig. 18:**
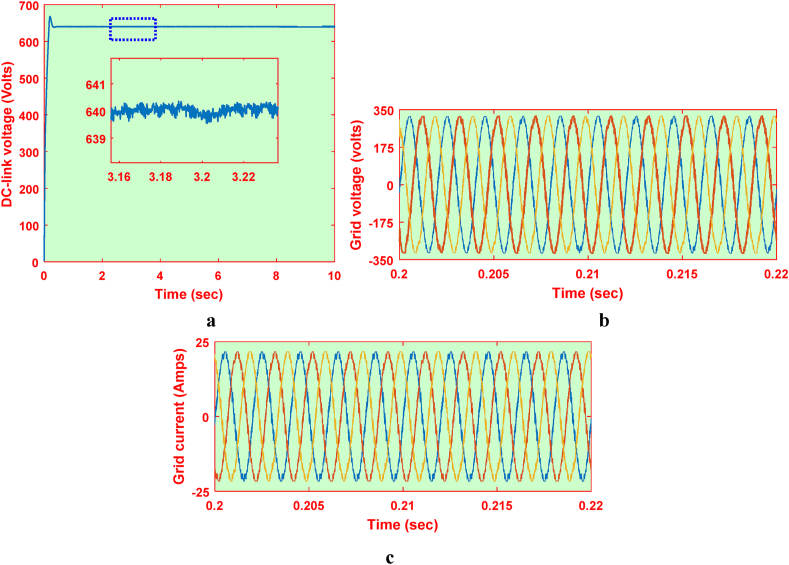
Grid parameters for proposed system (a) DC-link voltage, (b) Voltage and (c) Current.

**Fig. 19:**
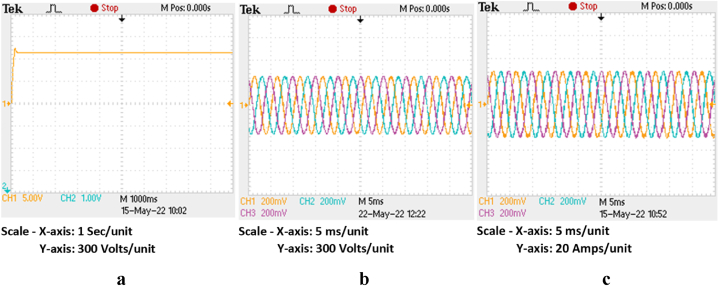
Grid parameters for proposed system in OPAL-RT (a) DC-link voltage, (b) Voltage and (c) Current.

The HRES is operated for the P&O scheme for both the wind and PV based systems. Also, the same system is operated for proposed IARV and SSM-ABC schemes for PV and wind systems respectively. The wind and PV profile are employed similar to Fig. 14, Fig. 16a. The respective DC link voltage for HRES with IARV and SSM-ABC schemes is represented in Fig. 18a. Also, the grid voltage and current for proposed system are represented in Figs. 18b and c respectively. Using OPAL-RT, the grid characteristics for P&O based system are represented in Fig. 19a, b & 19c.

**Fig. 20:**
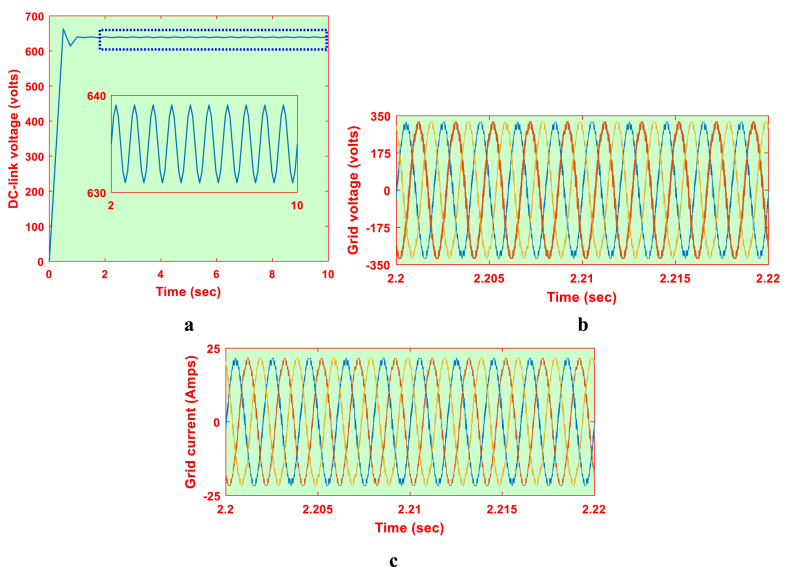
Grid parameters for P&O based system (a) DC-link voltage, (b) Voltage and (c) Current.

**Fig. 21:**
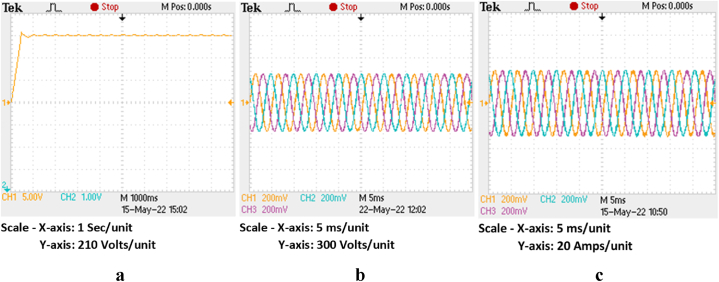
Grid parameters for P&O based system in OPAL-RT (a) DC-link voltage, (b) Voltage and (c) Current.

Similarly, the DC link voltage, grid voltage and current parameter for P&O based system are represented in Fig. 20a, b and 20c respectively. Using OPAL-RT, the grid parameters with P&O scheme are represented in Fig. 21a, b & 21c. Using the grid currents, the FFT analysis carried out for the considered system with P&O scheme and proposed schemes. The respective %fundamental components for frequency ranges from 0 to 100 Hz is represented in Table-6. The 50 Hz component is generally of 100%, it is opted out for better representation of inter-harmonics.

**Fig. 22:**
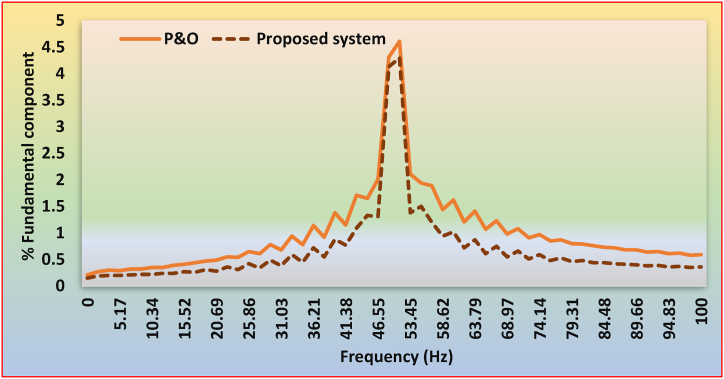
Comparison of inter-harmonic content of between P&O based system and proposed system.

**Fig. 23:**
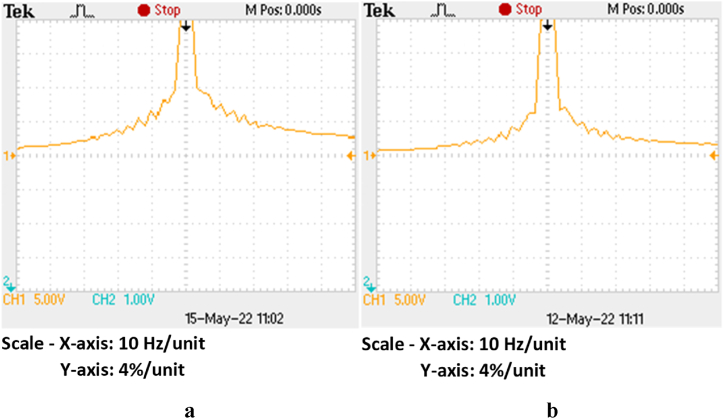
Inter-harmonics in grid current using OPAL-RT (a) P&O based system and (b) Proposed system.

The frequency versus %, Fig. 22 illustrates the basic elements for grid currents utilizing the P&O scheme and suggested MPPT approaches. Fig. 23a shows the FFT findings for the OPAL-RT system using the P&O scheme. Similarly, Fig. 23b depicts the OPAL-RT output for the suggested system. These results show that, in comparison to P&O-based systems, the system with proposed MPPT schemes exhibits low inter-harmonic content. Overall, compared to a P&O-based system, the suggested system exhibits a lower average inter-harmonic content of 32.19%.

## 5 Conclusion

In this paper, the DFIG, PV and BES are operated collectively to supply the reliable power to grid. Also, the proposed system is operated with proposed MPPT schemes for PV and WECS in generating improved dynamic characteristics. Further, the proposed MPPT schemes are operated to reduce the injection of inter-harmonics into the grid.

• An IARV MPPT scheme has been developed for addressing the drawback of ARV method.
• In comparison to P&O and MP&O algorithms, the suggested IARV MPPT has a greater efficiency of 99.09% for detecting PV MPP power [29].
• The suggested IARV MPPT method does not expnerience drift when irradiation changes abruptly, unlike P&O. Additionally, IARV MPPT scheme has a 14.5 ms shorter settling time than MP&O.
• The proposed IARV method displays better tracking efficiency at lower irradiation levels compared to CV MPPT.
• Moreover, IARV method does not require memory for tracking MPP. This leads to low processing cost compared to ARV scheme.
• SSM-ABC method is developed for DFIG MPPT to avoid the disadvantages of LS-P&O and SS-P&O algorithms.
• The proposed SSM-ABC scheme able to display the lower tracking time (<350 ms) compared to SS-P&O (>1300 ms). Also, it displays low steady state error of (0.001 rad/s) relative to LS-P&O algorithm (1.2 rad/s) [36].
• The SSM-ABC method exhibits the enhanced efficiency of 92.12% related to P&O based techniques.
• The suggested system uses the IARV and SSM-ABC schemes; it should be noted that this allows it to display less inter-harmonic material than P&O-based systems.
• It is noted that the proposed system shows the reduced average inter-harmonic content of 32.19% with respect to P&O scheme.
• The converter topology has been studied already with the varying irradiation and wind speed cases resulting in sudden change in input voltage case. Fault condition is not considered in this paper, main focus is driven towards the MPPT schemes with respect to the rapid variation in wind speed and solar irradiation. The fault condition along with the partial shading situation in solar panels will be considered for future work.

## Funding statement

No funding was supported for this research work.

## Data Availability Statement

No data available to this article.

## CRediT authorship contribution statement

**Boni Satya Varun Sai:** Writing – review & editing, Writing – original draft, Software, Formal analysis, Data curation, Conceptualization. **Rupali Mohanty:** Visualization, Validation, Methodology, Investigation, Formal analysis, Conceptualization. **Satyajit Mohanty:** Methodology, Investigation, Funding acquisition, Formal analysis, Data curation. **Debashis Chatterjee:** Writing – review & editing, Validation, Resources, Investigation, Formal analysis. **C. Dhanamjayulu:** Visualization, Validation, Supervision, Resources, Project administration, Investigation. **Ravikumar Chinthaginjala:** Writing – review & editing, Writing – original draft, Validation, Resources, Methodology. **Hossam Kotb:** Software, Resources, Methodology, Investigation, Funding acquisition. **Ali Elrashidi:** Visualization, Validation, Software, Funding acquisition, Data curation.

## Declaration of competing interest

The authors declare that they have no known competing financial interests or personal relationships that could have appeared to influence the work reported in this paper.

## Figures and Tables

**Table:** 

**Nomenclature**		
Photovoltaic	**:**	PV
Doubly fed induction generator	**:**	DFIG
Searching space minimization-based artificial bee colony	**:**	SSM-ABC
Maximum power point tracking	**:**	MPPT
Wind energy conversion system	**:**	WECS
Improved adaptive reference voltage	**:**	IARV
Maximum power point	**:**	MPP
Perturb and observe	**:**	P&O
Hybrid renewable energy system	**:**	HRES
Particle swarm optimization	**:**	PSO
Binary particle swarm optimization	**:**	BPSO
Modified perturb and observe	**:**	MP&O
Artificial neural network	**:**	ANN
Constant voltage	**:**	CV
Adaptive reference voltage	**:**	ARV
Permanent magnet synchronous generator	**:**	PMSG
Robust Perturbation Observer based Fractional Order Sliding Mode Controller	**:**	RPO-FOSMC
Grouped grey wolf optimizer	**:**	GGWO
Large step perturb and observe	**:**	LS-P&O
Small step perturb and observe	**:**	SS-P&O
Adaptive feedback linearization controller	**:**	FLC-A
Cuckoo search	**:**	CS
Artificial bee colony	**:**	ABC
Rotor side converter	**:**	RSC
Grid side converter	**:**	GSC
Photo current	**:**	I_p_
Diode	**:**	d
Solar panel output current	**:**	I_PV_
Solar panel output power	**:**	P_PV_
Series resistance	**:**	R_se_
Parallel resistance	**:**	R_pa_
Solar panel voltage	**:**	V_PV_
Switching pulse to converter	**:**	S, S_a_, S_b_
Wind turbine	**:**	WT
Wind turbine power	**:**	P_WT_
Battery voltage	**:**	V_BAT_
Battery current	**:**	I_BAT_
Reference battery current	**:**	I_BAT_*
Battery power	**:**	P_BAT_
Battery energy storage	**:**	BES
Grid power	**:**	P_Grid_
Grid current	**:**	I_gabc_
Grid voltage	**:**	V_gabc_
Stator voltage and current	**:**	V_abcs_, I_abcs_
Rotor voltage	**:**	V_abcr_
Mechanical power	**:**	P_m_
Rotor speed	**:**	W_m_
Reference rotor speed	**:**	W_m_*
DC-link voltage	**:**	V_dc_
Irradiation	**:**	G
Temperature	**:**	T
Wind speed	**:**	V_W_
Resistance	**:**	R_f_
Inductance	**:**	L_f_
Pole pair	**:**	p
Stator resistance	**:**	R_s_
Rotor resistance	**:**	$R_{r}$
Stator leakage inductance	**:**	$L_{\sigma s}$
Magnetizing inductance	**:**	$L_{m}$
Rotor leakage inductance	**:**	$L_{\sigma r}$
Turns ratio	**:**	u
Blade radius	**:**	R
Air density	**:**	ρ
Damping Coefficient	**:**	D
Inertia constant	**:**	H_m_
Optimal Tip speed ration	**:**	$\lambda_{opt}$
Maximum power coefficient	**:**	$C_{p\_\max}$
Unknown coefficients	**:**	C_1_–C_7_
MPP Power	**:**	P_M_
MPP Voltage	**:**	V_M_
MPP Current	**:**	I_M_
Short Circuit Current	**:**	I_sho_
Open Circuit Voltage	**:**	V_ope_
Temperature coefficient of I_sho_	**:**	α
Temperature coefficient of V_ope_	**:**	$T_{\beta }$
Rated battery capacity	**:**	C_Bat_
Curve fitting factor	**:**	A
Boltzmann constant	**:**	k
Electron charge	**:**	q
Saturation current of diode	**:**	I_o_
Standard testing condition	**:**	STC
Short circuit current at STC	**:**	I_sho,ref_
Open circuit voltage at STC	**:**	V_ope,ref_
Temperature at STC condition	**:**	T_ref_
Irradiation at STC	**:**	G_ref_
irradiation correction factor of V_ope_	**:**	*a*
MPP current	**:**	I_M_
MPP voltage	**:**	V_M_
MPP power	**:**	P_M_
Pitch angle	**:**	β
Air density	**:**	ρ
Power coefficient of a wind turbine	**:**	$C_{p}$
Swept area	**:**	*A*_*r*_
Tip speed ratio	**:**	λ
dq components of stator voltages and rotor voltages	**:**	$V_{ds}$, $V_{qs}$, $V_{dr}$, $V_{qr}$
dq components of stator flux and rotor flux	**:**	$\psi_{ds}$, $\psi_{qs}$, $\psi_{dr}$, $\psi_{qr}$
Rotor current	**:**	$i_{r}$
Rotor speed	**:**	$\omega_{r}$
Synchronous speed	**:**	$\omega_{s}$
Electromagnetic torque	**:**	$T_{em}$
dq components of stator and rotor currents	**:**	$i_{ds}$, $i_{qs}$, $i_{dr}$, $i_{qr}$
Stator flux	**:**	$\psi_{s}$
Rotor flux	**:**	$\psi_{r}$
Proportional-integral controller	**:**	PI
Power-voltage	**:**	P–V
Current-voltage	**:**	I–V
Series- Parallel configuration	**:**	S–P
constants	**:**	a, b, K_1_, K_2_, ∈, γ
Single panel	**:**	1S
MPP voltage for 1S configuration at irradiation G	**:**	${\left({V_{M}|}_{1S}\right)}_{G}$
MPP voltage for 24S–2P configuration at irradiation G	**:**	${\left({V_{M}|}_{24S-2P}\right)}_{G}$
MPP voltage for 1S configuration at STC	**:**	${\left({V_{M}|}_{1S}\right)}_{STC}$
Open circuit voltage for 1S configuration at STC	**:**	${\left({V_{ope}|}_{1S}\right)}_{STC}$
MPP current for 1S configuration at irradiation G	**:**	${\left({I_{M}|}_{1S}\right)}_{G}$
MPP current for 24S–2P configuration at irradiation G	**:**	${\left({I_{M}|}_{24S-2P}\right)}_{G}$
MPP current for 1S configuration at STC	**:**	${\left({I_{M}|}_{1S}\right)}_{STC}$
Short circuit current for 1S configuration at STC	**:**	${\left({I_{sho}|}_{1S}\right)}_{STC}$
Model reference adaptive system	**:**	MRAS
Number of bees	**:**	Np
Maximum rotor speed	**:**	$\omega_{\max}$
Fast Fourier Transform	**:**	FFT
Minimum rotor speed	**:**	$\omega_{\min}$
Optimum rotor speed	**:**	$\omega_{opt}$
Food source position with high nectar amount	**:**	X_hi_
